# Obstetric violence: reflection on reporting to achieve sustainable development goals

**DOI:** 10.1590/0034-7167-2024-0523

**Published:** 2025-07-11

**Authors:** Emmanuele Mainart Ildefonso, Laura Christina Macedo, Tatiane Herreira Trigueiro, Isabele Melo Martins

**Affiliations:** I Universidade Federal do Paraná. Curitiba, Paraná, Brazil; II Empresa Brazileira de Serviços Hospitalares, Maternidade Escola Assis Chateaubriand. Fortaleza, Ceará, Brazil

**Keywords:** Violence, Obstetric Violence, Disease Notification, Sustainable Development, Gender-Based Violence, Violencia, Violencia Obstétrica, Notificación, Desarrollo Sostenible, Violencia de Género

## Abstract

**Objectives:**

to explain obstetric violence, its consequences, and the importance of mandatory reporting to achieve the Sustainable Development Goals.

**Methods:**

a reflective study based on references and the researchers’ expertise on the subject.

**Results:**

the first part of the results presents the characterization of violence and its main consequences. The second part reflects on how to record obstetric violence on the Individual Reporting Form for Interpersonal and Self-Inflicted Violence, even though there is no specific field for recording this violence.

**Final Considerations:**

underreporting cases of obstetric violence negatively compromises the achievement of the Sustainable Development Goals - specifically with regard to goal 5 “Gender Equality” and goals 3 “Health and Well-being” and 10 “Reduced Inequalities”, but it is possible to report. Reporting is essential for recognizing cases and implementing preventive actions.

## RESUMEN

**Objetivos:**

explicar la violencia obstétrica, sus consecuencias y la importancia de la notificación obligatoria para alcanzar los Objetivos de Desarrollo Sostenible.

**Métodos:**

estudio reflexivo basado en referencias y experticia de los investigadores sobre el tema.

**Resultados:**

la primera parte de los resultados presenta la caracterización de la violencia y las principales consecuencias. La segunda parte reflexiona sobre cómo registrar la violencia obstétrica en el Formulario de Denuncia Individual de Violencia Interpersonal y Autoinfligida, aunque no exista un campo específico para registrar esta violencia.

**Consideraciones Finales:**

el subregistro de los casos de violencia obstétrica compromete negativamente el logro de los Objetivos de Desarrollo Sostenible - específicamente en lo que respecta al objetivo 5 “Igualdad de género” y los objetivos 3 “Salud y bienestar” y 10 “Reducción de las desigualdades”, pero es posible denunciarlos. La notificación es esencial para reconocer casos e implementar acciones preventivas.

## INTRODUCTION

Violence against women is a global public health problem. It is violence applied with the intent to dominate and exploit women, treating a human being as an object rather than a subject, based on biological sex differences. It is present in the daily lives of various societies and is often perceived and accepted as part of the established order, which contributes to the invisibility of the problem. Any violence against women is gender-based violence, being a frequent practice in a society that violates women because of their gender identity and their female condition^(1)^.

Women are disrespected and suffer prejudice constantly, making being born female enough to designate a life of struggle and constant injustices. Among the various types of violence committed against women, obstetric violence (OV) is any violent act perpetrated against pregnant, laboring, or postpartum women during obstetric care. It is characterized by neglect in care, discrimination, verbal violence, rough treatment, threats, reprimands, shouting, humiliation, physical, psychological, and sexual violence, as well as the use of technologies, interventions, and unnecessary procedures without plausible justification during obstetric care^(1)^.

OV can occur both in interpersonal relationships, coming from individuals within or outside the family circle, and in institutional settings, where the violence is committed by professionals providing care to this population. In institutional settings, the most frequent forms of violence involve the appropriation of women’s bodies and interventions without their free and informed consent, disregarding their decisions about their bodies and sexuality, as well as the abuse of professional power over women in its various forms^(2)^.

In 2015, the countries that are part of the United Nations (UN) were presented with a sustainable development agenda for the next 15 years. The 2030 Agenda consists of 17 Sustainable Development Goals (SDGs) that seek to “ensure human rights, end poverty, fight inequality and injustice, achieve gender equality and the empowerment of women and girls, act against climate change, as well as address other major challenges of our time”. In pursuit of equity and the confrontation of all forms of violence against women, SDG 5 - Gender Equality - includes as one of its targets the need for strategies to promote, protect, and guarantee women’s sexual and reproductive rights. Therefore, it is undeniable that achieving this goal involves combating OV. In addition to its direct relationship with achieving gender equality, combating OV also influences the achievement of other SDGs: 3 - “Good Health and Well-being” and 10 - “Reduced Inequalities”^(2)^.

In 2015, the countries that are part of the United Nations (UN) were presented with a sustainable development agenda for the next 15 years. The 2030 Agenda consists of 17 Sustainable Development Goals (SDGs) that seek to “ensure human rights, end poverty, fight inequality and injustice, achieve gender equality and the empowerment of women and girls, act against climate change, as well as address other major challenges of our time”. In pursuit of equity and the confrontation of all forms of violence against women, SDG 5 - Gender Equality - includes as one of its targets the need for strategies to promote, protect, and guarantee women’s sexual and reproductive rights. Therefore, it is undeniable that achieving this goal involves combating OV. In addition to its direct relationship with achieving gender equality, combating OV also influences the achievement of other SDGs: 3 - “Good Health and Well-being” and 10 - “Reduced Inequalities”^(2)^.

In Brazil, since the 1970s, the feminist movement has advocated for gender equality. In the 1990s, women’s groups involved in the development of the National Policy for Comprehensive Women’s Health Care^(3)^ ensured the inclusion of obstetric care provisions aimed at protecting women’s rights in both reproductive planning and the humanized care of pregnancy, childbirth, and the postpartum period. Subsequently, additional guidelines were introduced to regulate these services, such as the Humanization of Labor and Birth Program^(3)^ and the Stork Network (*Rede Cegonha*). It is important to emphasize that all of these initiatives align with the achievement of the SDGs.

In 2019, as part of the research “Meanings of Birth”, which emerged from the “Meanings of Birth” exhibition, a study was conducted focusing on the perception of OV. Among the 555 women who visited the exhibition during pregnancy, OV was reported by 12.6% of them and was associated with factors such as marital status, low income, and lack of a partner. The main forms of violence reported were related to lithotomy position during delivery, the use of Kristeller’s maneuver, and the separation of the mother and baby immediately after birth without explicit necessity. It is believed that this figure is underestimated, pointing to a lack of knowledge and misinformation among women about safe care recommendations, as well as what constitutes abusive and scientifically unsupported practices^(4-6)^.

The abuse of power by healthcare professionals over the women they care for is recurrent, as they attempt to use their professional authority to enforce practices and/or techniques that are not in line with current protocols and scientific evidence, to the detriment of the desires and needs of the women being cared for, who are in a vulnerable position. The desires and wishes of women are not taken into account, and they end up experiencing situations they did not want and unnecessary interventions. In these situations, their bodies and voices are not properly respected^(6)^.

OV often goes unnoticed. Whether subtle or intense, it can demoralize and influence the choices of the person being harassed. Moral conduct overrides ethical conduct, resulting in value judgments that are often verbalized as: “See you again next year!”, “At this rate, you’ll have a soccer team!”, “Isn’t it time to stop?”, “Brave of you to get pregnant at this age”, “You got pregnant so young, you ruined your life!”, “You got pregnant, now deal with it!”. At times, these remarks lead to feelings of insecurity, fear, and influence over future decisions, caused by professionals who should be providing support and care^(3)^.

OV practices permeate all levels of healthcare - Primary, Secondary, and Tertiary. They occur in both private and public services, from the discovery of pregnancy onward. At times, women are violated at the start of prenatal care, when healthcare professionals underestimate women’s agency by making unnecessary remarks at this stage, to after childbirth, downplaying pain and disregarding the privacy and autonomy of postpartum women^(6)^.

By recognizing OV as an existing issue that needs to be addressed, the next important step is to reflect on the care practices offered by healthcare professionals. Nursing professionals are often recognized for advocating for patients, but at times, they may also be in the position of perpetrating violence.

Considering the urgency of fostering discussions on the topic of OV, its consequences, and ways to combat it, this article aims to reflect on the following question: What is the relationship between reporting OV and the SDGs?

## OBJECTIVES

To reflect on how OV manifests and the importance of mandatory reporting for achieving gender equality.

## METHODS

This is a theoretical-reflective essay, referred to as a “reflection”. Although free from methodological formalities, reflections are representations free from prejudices and paradigms, enabling readers to adopt a participatory stance, being prompted to draw their own conclusions and enhance their knowledge.

The authors delved into national and international studies on OV, documents, guidelines, legislation, and regulations that could enrich the debate between everyday practice and theory. The main discussion points were divided into two parts. The first presents the characterization of OV and its main consequences. The second part discusses how to report OV in the Individual Notification Form for Interpersonal and Self-Inflicted Violence, even though there is no specific field for reporting this type of violence.

Ethical review and approval were waived for this study, as it is a theoretical-reflective essay. However, ethics will be ensured through loyalty to the information addressed in the article.

## DISCUSSION

### The impact of the appropriation of childbirth by hospital technocracy and the normalization of obstetric violence among healthcare professionals

Women in the gravid-puerperal cycle experience not only changes in their bodies but also abrupt changes in their lives. In addition to hormonal and physiological changes, other experiences can exacerbate the fragility and vulnerability experienced during this period. Lack of a support network, the end of a relationship, abandonment by the father regarding paternal responsibilities, unemployment, and the onset of illnesses are some conditions that can negatively affect women’s physical and mental health during this time. However, in healthcare services, many women experience OV, unfortunate experiences caused by professionals who should provide support and care, contributing to lifelong trauma as they feel attacked, violated, and disrespected. Institutional OV encompasses all forms of disrespect, exposure, unnecessary interventions, discrimination, lack of supplies, and misuse of power by healthcare professionals. As a consequence, the loss of autonomy and freedom of choice during the gravid-puerperal cycle directly impacts women’s physical and mental health^(4)^.

OV gained momentum as hospitals became recognized as the primary space for childbirth. This reality led to the valorization of numerous interventions but also devalued women’s autonomy and strength. The technocratic model began to prioritize medical procedures over women’s needs, resulting in the inappropriate and unnecessary use of technologies that contradict best practices in childbirth care, exacerbating violence and disregarding women’s capacity and strength during labor^(4)^.

The technocratic model of care for women in the gravid-puerperal cycle reinforces the protagonism of healthcare professionals, particularly those specialized in gynecology and obstetrics. In this perspective, women become secondary actors, relegated to observation and submission. In this scenario, it is difficult to recognize the OV(s) that routinely occur as “part” of the hospital process. Certain groups of women are at higher risk of experiencing OV: Black and Brown women, adolescents, women with lower levels of education, from lower economic classes, without a partner or companion, those engaged in unpaid work, those receiving care in public health services, women from the North and Northeast regions of Brazil, multiparous women, women dissatisfied with their current pregnancy, or those who attempted to terminate their pregnancy^(5)^. These groups are precisely those considered priorities for implementing actions in support of the SDGs.

Even so, OV is still seen as taboo, often concealed by those who should be working toward its eradication. In 2019, supported and motivated by representatives of the Federal Council of Medicine (CFM in Portuguese) and gynecology societies, the Ministry of Health (MS in Portuguese) attempted to ban the term “Obstetric Violence”, forcing its replacement with “inadequate care” or “poor practice”, arguing that the term obstetric violence is inconsistent with a honorable professional activity aimed at protecting individuals and saving lives^(6)^.

The CFM argued that a terminology identifying a professional activity as criminal should not be used, favoring instead the term “poor practice”, as is the case in other areas of medicine. The Brazilian Federation of Gynecology and Obstetrics Associations (FEBRASGO in Portuguese) further emphasized that procedures such as episiotomy, use of oxytocin, cesarean sections, among others, should not be generalized as “villains”, as they are medical procedures used to reduce maternal and infant mortality and protect against worse complications^(7)^. However, after women’s advocacy groups reacted against the removal of the term, the Federal Public Ministry (MPF in Portuguese) opposed the MS’s decision, challenging the justification and maintaining the use of the term OV^(6)^.

Women also face difficulty in recognizing OV, as there is a gap between acknowledging that they have suffered abuse and labeling it as such. OV is a violation of the body, dignity, and autonomy of women, like any other violence committed against women, with the difference being that it occurs during the reproductive phase^(5,8)^. Therefore, the types of OV include:

**Psychological violence:** Women suffer from feelings of inferiority, vulnerability, abandonment, loss of their rights, and violation of their dignity and autonomy (deception, mockery, and humiliation)^(5)^.

**Physical violence:** Any procedure that causes pain or physical harm without evidence-based recommendations. Examples include food deprivation, restriction of movement, elective cesarean sections without clinical indication, and Kristeller’s maneuver^(5)^.

**Sexual violence:** Violation of intimacy or modesty, affecting the sense of sexual and reproductive integrity, with or without access to sexual organs and intimate parts of the body. Examples include episiotomy, invasive, frequent, or aggressive vaginal exams, enemas, and cesarean sections without informed consent^(5)^.

**Institutional violence:** Actions or organizational structures that hinder, delay, or prevent women’s access to their established rights, such as omitting or violating women’s rights during pregnancy, childbirth, and the postpartum period, denying access to healthcare services, preventing breastfeeding without clinical justification, and prohibiting the presence of a companion of their choice^(5)^.

**Material violence:** Active or passive actions and conduct aimed at obtaining financial resources from women in reproductive processes, violating their legally guaranteed rights for the benefit of individuals or organizations. This includes improper charges by health plans and professionals, and inducing women to purchase private health plans under the argument that it is the only way to ensure a companion^(5)^.

**Media violence:** Actions carried out by professionals through communication channels aimed at psychologically violating women in reproductive processes; advocacy of scientifically contraindicated practices for social, economic, or dominance purposes. Examples include promoting cesarean sections for non-scientific reasons, ridiculing vaginal birth, marketing formula milk at the expense of breastfeeding, and encouraging early weaning^(5)^.

In addition to the power imbalance between professionals and women, which can interfere with women’s autonomy and the preservation of their physical and psychological integrity, the recognition of women’s rights to informed choice and refusal, and to not be subjected to non-consensual interventions, is recent and has not yet become part of the culture of either professionals or women. The main consequences of OV reported are depression, fear of repeating the experience, physical complications, and maternal and infant mortality^(5,8)^.

In this context, nursing, as a profession grounded in care and actively involved in all phases of care for women in the gravid-puerperal cycle, is essential for recognizing and acting to reduce OV^(8)^.

### Reporting - Recognizing to Act

Decree No. 5,099 of 2004 regulated the mandatory reporting of cases of violence against women nationwide, assigning the MS the coordination of the strategic action plan for the establishment of sentinel reference services. That same year, MS/GM Ordinance No. 2,406 established the mandatory reporting service for violence against women and approved the instrument, protocols, and flow for reporting in public and private health services^(9)^.

In 2016, the MS made available the “VIVA” Guidelines for completing the Notification Form for Interpersonal and Self-Inflicted Violence. The aim was to support professionals working in reporting units/services to standardize the completion of this data collection tool, based on a set of variables and categories that depict violence perpetrated against population groups. The guidelines emphasize the legal requirement to report cases of violence against women, including OV^(10)^.

However, despite the obligation of healthcare professionals or those responsible for public and private services to report, one of the concerns expressed by public health policies is the lack of records of cases of violence against women in health services. Furthermore, the Individual Notification Form (FIN in Portuguese) does not have a specific field to identify the individual who committed the violent act. Unlike a police report, whose purpose is to initiate a criminal investigation, the data recorded about the probable aggressor are intended to generate epidemiological data that serve as the basis for prevention and tracking strategies, focusing on higher-risk scenarios^(10)^.

**Figure 1:**
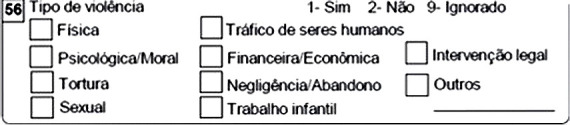
Field 56

**Figure 2:**
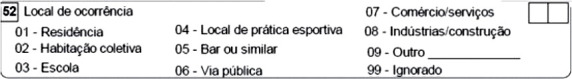
Field 52

**Figure 3:**
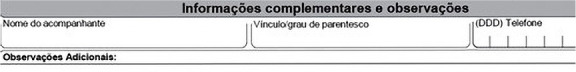
Additional Information

*Source: VIVA - Guidelines for Notification of Interpersonal and Self-Inflicted Violence (2024).*

Reporting the incident has nothing to do with filing a complaint or other police or legal procedures. Reporting is part of the Comprehensive Care Pathway for Children, Adolescents, and Their Families in Situations of Violence, which includes welcoming, providing care, promoting preventive measures, treatment, follow-up within the care network, and social protection, in addition to surveillance, violence prevention, and the promotion of health and a culture of peace^(10)^. The FIN does not have a specific field for identifying OV cases; however, field 56 of the FIN (Figure 1) allows for the recording of various types of aggression. In field 52 (Figure 2), it is possible to record the location of the incident as “09” (other), and details related to the gravid-puerperal cycle can be noted in the space reserved for additional information (Figure 3)^(10)^.

Given the above, for the information collected through notifications to aid in the planning and implementation of effective public policies to address violence, and to ensure the complete and accurate completion of the notification form, it is essential that the information be valid, up-to-date, and comprehensive, and that professionals have knowledge of the types of violence and the correct identification of suspected cases. These data and information are essential for the quality of care provided by healthcare teams, the management of healthcare units and services, the planning of health actions and policies, and for the production of knowledge and social control, as reporting violence constitutes the first step in the path toward controlling this issue^(8)^.

Furthermore, we know that merely recognizing OV as a challenge to be addressed does not conclude the discussion. Reporting OV is just one step among many necessary actions to reduce all forms of violence against women. The solution depends on various strategies, such as health education initiatives^(8)^. An example of such an initiative is the Massive Open Online Course (MOOC) designed to train healthcare professionals on OV, developed as part of the Professional Master’s Program at Federal University of Paraná (UFPR), to be made available to workers at the *Hospital de Clínicas* Complex of the UFPR, managed by the Brazilian Company of Hospital Services.

## FINAL CONSIDERATIONS

All women have the right to a childbirth free from violence, with respect, informed assistance and choice, and practices based on the best scientific evidence. It is necessary to talk about OV, its risks, and to report it in order to break away from this form of care that places women in a position of inferiority, submission, and fails to provide safe and humanized care.

Reflections are not conclusive; on the contrary, they are starting points for analyzing topics that can be explored and debated from various perspectives. In this reflection, we consider that, to achieve gender equality, the main goal of SDG 5, the reporting of OV must be widely studied and discussed. Just as reporting has been an important tool for recognizing other forms of violence against women, it should also be used as a tool for recognizing OV.

The underreporting of OV cases directly and negatively impacts the achievement of the SDGs - specifically Goal 5, “Gender Equality”, but also Goals 3, “Good Health and Well-being”, and 10, “Reduced Inequalities”.

Nursing professionals who provide obstetric care must do so in a safe and humanized manner, free from invasive and unnecessary practices. They must provide education to pregnant women and their families on this topic, their rights, and tools for reporting violations of these rights. It is hoped that this article will contribute to nursing professionals who care for women in the gravid-puerperal cycle seeking to understand the reporting of OV cases. Reporting is a way to give voice to women who silently suffer from institutionalized violence that often goes unnoticed.
