# ERRATUM

**DOI:** 10.1590/0034-7167.20257806e09

**Published:** 2025-12-08

In the article “Obstetric violence: reflection on reporting to achieve sustainable development goals”, with DOI number: https://doi.org/10.1590/0034-7167-2024-0523, published in Revista Brasileira de Enfermagem, 2025;78(3):e20240523, in the authorship:

Where it read:

**Isabele Melo Martins^II^**

**ORCID: 0000-0002-4455-0499**

It reads:

**Isabelle Melo Martins^II^**

**ORCID: 0000-0002-4455-0499**

