# Supplementary material for: Obstetric violence: reflection on reporting to achieve sustainable development goals
Source: Rev Bras Enferm. 2025 Jul 11;78(3):e20240523. doi: 10.1590/0034-7167-2024-0523 (PMC12252925; doi:10.1590/0034-7167-2024-0523)
Supplement: Translation

# Translation: Violência obstétrica: reflexão sobre a notificação para alcance dos objetivos de desenvolvimento sustentável

**DOI:** 10.1590/0034-7167-2024-0523pt

**Authors:** Emmanuele Mainart Ildefonso, Laura Christina Macedo, Tatiane Herreira Trigueiro, Isabele Melo Martins

**Affiliations:** I Universidade Federal do Paraná. Curitiba, Paraná, Brasil; II Empresa Brasileira de Serviços Hospitalares, Maternidade Escola Assis Chateaubriand. Fortaleza, Ceará, Brasil

**Keywords:** Violência, Violência Obstétrica, Notificação, Desenvolvimento Sustentável, Violência de Gênero

## Abstract

**Objetivos:**

explanar sobre violência obstétrica, suas consequências e importância da notificação compulsória para alcance dos Objetivos de Desenvolvimento Sustentável.

**Método:**

estudo reflexivo embasado em referências e na expertise das pesquisadoras sobre a temática.

**Resultados:**

a primeira parte dos resultados apresenta a caracterização da violência e as principais consequências. Na segunda parte reflete-se sobre como registrar a violência obstétrica na Ficha Individual de Notificação de Violência Interpessoal e Autoprovocada mesmo não havendo campo específico para registro dessa violência.

**Considerações Finais:**

a subnotificação dos casos de violência obstétrica compromete negativamente o alcance dos Objetivos de Desenvolvimento Sustentável - especificamente no que refere ao objetivo 5 “Igualdade de Gênero” e aos objetivos 3 “Saúde e Bem-estar”, 10 “Redução das Desigualdades”, porém é possível notificar. A notificação é imprescindível para reconhecimento dos casos e implementação de ações preventivas.

## INTRODUÇÃO

A violência contra a mulher é um problema mundial de saúde pública. É uma violência aplicada com o intuito de dominar e explorar mulheres, tratando um ser humano como objeto e não como sujeito, com base na diferença biológica de sexo. Está presente no cotidiano das diversas sociedades e, muitas vezes, é percebida e aceita como parte da ordem estabelecida, o que contribui para a invisibilidade do problema. Qualquer violência contra a mulher é uma violência de gênero, sendo uma prática frequente em uma sociedade que violenta as mulheres pela sua identidade de gênero e por sua condição feminina^(1)^.

As mulheres são desrespeitadas e sofrem preconceito constantemente, tornando o nascer mulher o suficiente para designar uma vida de luta e de constantes injustiças. Dentre os vários tipos de violências cometidas contra a mulher, a violência obstétrica (VO) é qualquer ato violento praticado contra gestantes, parturientes ou puérperas durante a assistência obstétrica. Caracteriza-se por negligência na assistência, discriminação, violência verbal, tratamento grosseiro, ameaças, repreensão, gritos, humilhação, violência física, psicológica, sexual, além do uso sem indicação plausível de tecnologias, intervenções e procedimentos desnecessários durante a assistência obstétrica^(1)^.

A VO pode ocorrer tanto nas relações interpessoais, vinda de pessoas do ciclo intra ou extrafamiliar, quanto no ambiente institucional, onde a violência é cometida por profissionais que prestam assistência a esse público. No ambiente institucional, as violências mais frequentes referem-se à apropriação do corpo da mulher e a intervenções sem o consentimento livre e esclarecido da mesma, menosprezando as decisões sobre seu corpo e sexualidade, além do abuso do poder profissional sobre a mulher nas suas diversas formas^(2)^.

Em 2015, aos países que fazem parte da Organização das Nações Unidas (ONU), foi proposta uma agenda de desenvolvimento sustentável para os próximos 15 anos. A Agenda 2030 é composta por 17 Objetivos de Desenvolvimento Sustentáveis (ODS) que buscam “assegurar os direitos humanos, acabar com a pobreza, lutar contra a desigualdade e a injustiça, alcançar a igualdade de gênero e o empoderamento de mulheres e meninas, agir contra as mudanças climáticas, bem como enfrentar outros dos maiores desafios de nossos tempos”. Em busca da equidade e confrontação de todas as formas de violência contra a mulher, o ODS 5 - Igualdade de Gênero - apresenta como uma das metas a necessidade de estratégias para a promoção, proteção e garantia dos direitos sexuais e reprodutivos das mulheres. Portanto, é inegável que o alcance desse objetivo perpassa pelo combate à VO. Além da relação direta com o alcance da igualdade de gênero, o combate à VO tem influência no alcance de outros ODS: 3 - “Saúde e Bem-estar” e 10 - “Redução das Desigualdades”^(2)^.

No Brasil, desde 1970, o movimento feminista tem lutado pela igualdade de gênero. Na década de 1990, grupos de mulheres que participaram da formulação da Política Nacional de Atenção Integral à Saúde da Mulher^(3)^ garantiram que cuidados relacionados ao atendimento obstétrico, com vistas a assegurar os direitos da mulher tanto em relação ao planejamento reprodutivo quanto na atenção humanizada à gravidez, ao parto e ao puerpério, estivessem previstos. Posteriormente, outras diretrizes vieram regulamentar esses cuidados, como o Programa de Humanização do Parto e Nascimento^(3)^ e a Rede Cegonha. Cabe ressaltar que todas convergem para o alcance dos ODS.

Em 2019, como parte da pesquisa “Sentidos do Nascer”, surgida da exposição Sentidos do Nascer, foi realizado um estudo com enfoque na percepção sobre VO. Das 555 mulheres que visitaram a exposição durante a gestação, a VO foi reportada por 12,6% delas e associada a fatores como: estado civil, baixa renda e ausência de companheiro. As principais violências relatadas estão relacionadas ao parto em posição litotômica, à realização da manobra de Kristeller e à separação da mãe e do bebê logo após o parto sem expressa necessidade. Acredita-se que esse valor esteja subestimado, o que aponta para o desconhecimento e a desinformação das mulheres sobre as recomendações assistenciais seguras, bem como sobre o que são práticas abusivas e sem respaldo científico^(4-6)^.

É recorrente o abuso de poder de profissionais da saúde sobre a mulher que está sendo assistida, tentando utilizar a posição de poder profissional para fazer valer práticas e/ou técnicas que não estão em conformidade com protocolos e evidências científicas atuais, em detrimento dos desejos e necessidades da mulher atendida, a qual se encontra em posição vulnerável. O desejo e a vontade da mulher não são levados em consideração, e essas acabam experimentando vivências que não desejavam e intervenções desnecessárias. Nessas situações, seu corpo e sua voz não são devidamente respeitados^(6)^.

A VO passa muitas vezes despercebida. De forma sutil ou intensa, pode desmoralizar e influenciar nas opções de escolha da pessoa assediada. A conduta moral se sobrepõe à conduta ética, acarretando em juízos de valor que, por muitas vezes, são verbalizados como: “Ano que vem te encontro novamente!”, “Desse jeito, vai ter um time de futebol!”, “Já está na hora de parar, não acha?”, “Corajosa, engravidar com essa idade”, “Engravidou tão nova, acabou com a sua vida!”, “Engravidou, agora aguenta!”. Por vezes, acarretam sentimentos de insegurança, medo e influência nas decisões futuras, ocasionados por profissionais que deveriam amparar e cuidar^(3)^.

Práticas de VO permeiam todos os níveis de atenção à saúde - Primária, Secundária e Terciária. Ocorrem em serviços privados e públicos, desde a descoberta da gestação. Por vezes, mulheres são violentadas no início do acompanhamento pré-natal, quando profissionais da saúde subestimam o protagonismo feminino, proferindo indagações desnecessárias para esse momento, até após o parto, menosprezando a dor e desrespeitando a intimidade e a autonomia da puérpera^(6)^.

Ao reconhecer a VO como uma situação posta e que precisa ser enfrentada, o próximo importante passo é refletir sobre as práticas de cuidado oferecidas por profissionais de saúde. Profissionais de enfermagem costumam ser reconhecidos por advogar pelas pacientes, mas, por vezes, podem estar na posição de perpetradores da violência.

Considerando a urgência de fomentar discussões sobre a temática da VO, suas consequências e formas de combatê-la, este artigo se propõe a refletir sobre a seguinte questão: Qual a relação entre a notificação da VO e os ODS?

## OBJETIVOS

Refletir sobre como se apresenta a VO e a importância da notificação compulsória para o alcance da Igualdade de gênero.

## MÉTODOS

Trata-se de um ensaio teórico-reflexivo, denominado pelo termo “reflexão”. Apesar de livre da formalidade metodológica, reflexões são representações isentas de preconceitos e paradigmas, que possibilitam ao leitor e à leitora uma postura de participante, sendo instigados e instigadas a tirar as suas próprias conclusões e aprimorar seus conhecimentos.

As autoras se debruçaram sobre estudos nacionais e internacionais acerca da VO, documentos, diretrizes, legislações e normas que possam enriquecer o debate entre a prática cotidiana e a teoria. Os principais pontos de discussão foram divididos em dois momentos. O primeiro apresenta a caracterização da VO e suas principais consequências. Na segunda parte, discute-se como registrar a VO na Ficha Individual de Notificação de Violência Interpessoal e Autoprovocada, mesmo não havendo campo específico para registro dessa violência.

A revisão ética e aprovação foram dispensadas para este estudo, por ser um estudo do tipo ensaio teórico-reflexivo. Todavia, será garantida a ética por meio da lealdade às informações abordadas no artigo.

## DISCUSSÃO

### O impacto da apropriação do parto pela tecnocracia hospitalar e naturalização da violência obstétrica entre profissionais de saúde

A mulher no ciclo gravídico-puerperal experimenta não só a modificação do seu corpo, mas de sua vida, de forma abrupta. Além das alterações hormonais e fisiológicas, outras experiências podem potencializar a fragilidade e a vulnerabilidade vivenciadas nesse período. Não possuir uma rede de apoio, enfrentar o término de um relacionamento, abandono por parte do genitor quanto às responsabilidades da paternidade, desemprego e surgimento de doenças são algumas condições que podem influenciar negativamente a saúde física e mental da mulher nesse período. Porém, nos serviços de saúde, muitas mulheres sofrem VO, experiências infelizes ocasionadas por profissionais que deveriam amparar e cuidar, corroborando com traumas carregados por uma vida, ao se sentirem agredidas, invadidas e desrespeitadas. A VO institucional é toda forma de desrespeito, exposição, intervenção desnecessária, discriminação, falta de insumos e uso indevido do poder pelos profissionais de saúde. E, como consequência, a perda da autonomia e liberdade de escolha durante o ciclo gravídico-puerperal reflete diretamente em forma de prejuízos na saúde física e mental da mulher^(4)^.

A VO foi ganhando força à medida que os hospitais passaram a ser reconhecidos como o principal espaço para se dar à luz. Essa realidade trouxe a valorização do uso de muitas intervenções, mas também desvalorizou a autonomia e a força feminina. O modelo tecnocrático passou a privilegiar a assistência direcionada ao trabalho médico e não às mulheres, culminando no uso indevido e desnecessário de tecnologias que vão contra as boas práticas de assistência ao parto, potencializando a violência e desconsiderando a capacidade e força da mulher no processo de parturição^(4)^.

O modelo tecnocrático de assistência à mulher no ciclo gravídico-puerperal fortalece o protagonismo do profissional de saúde, prioritariamente especializado em ginecologia e obstetrícia. A mulher, nessa perspectiva, torna-se atriz secundária, restando-lhe a observação e a submissão. Nesse cenário, é difícil reconhecer as VO(s) que acontecem rotineiramente como “parte” do processo hospitalar. Alguns grupos de mulheres apresentam maior risco de sofrerem VO: mulheres da cor da pele preta/parda, adolescentes, mulheres com menor escolaridade, de classe econômica mais baixa, sem companheiro/acompanhante, que possuem um trabalho não remunerado, atendidas em serviço público de saúde, oriundas das regiões Norte e Nordeste, multíparas, mulheres insatisfeitas com a gestação atual ou que tentaram interromper a gestação^(5)^, justamente grupos considerados prioritários para implantação de ações em prol dos ODS.

Mesmo assim, a VO ainda é vista como tabu, muitas vezes sendo ocultada por quem deveria trabalhar por sua erradicação. Em 2019, apoiado e motivado por representantes do Conselho Federal de Medicina (CFM) e pela sociedade de ginecologia, o Ministério da Saúde (MS) tentou impedir o uso do termo Violência Obstétrica, forçando a troca da expressão “violência obstétrica” por “assistência indevida” ou “má prática”, com a justificativa de que o termo violência obstétrica não é condizente com uma atividade profissional honrosa que visa à proteção do indivíduo e a salvar vidas^(6)^.

O CFM defendia que não poderia ocorrer a utilização de uma terminologia que identificasse uma atividade profissional como criminosa, ao invés de má prática, como acontece com as demais áreas da medicina. A Federação Brasileira das Associações de Ginecologia e Obstetrícia (FEBRASGO) ainda reforçou que procedimentos como episiotomia, uso de ocitocina, cesarianas, dentre outros, não poderiam ser generalizados como “vilões”, pois são procedimentos médicos utilizados para a redução da mortalidade materno-infantil, além da proteção contra piores complicações^(7)^. Porém, após grupos de defesa das mulheres reagirem contra a retirada do termo, o Ministério Público Federal (MPF) se posicionou contra a atitude do MS, confrontando a justificativa utilizada e mantendo o uso do termo VO^(6)^.

Também as mulheres apresentam dificuldade de reconhecimento da VO, pois há uma distância entre reconhecer que sofreu uma agressão e nominá-la como tal. A VO é uma forma de violação do corpo, da dignidade e da autonomia feminina, como qualquer outra violência cometida contra a mulher, o que a diferencia é que ela acontece na fase reprodutiva^(5,8)^. Portanto, são tipos de VO:

**Violência psicológica:** a mulher sofre com sentimentos de inferioridade, vulnerabilidade, abandono, perda dos seus direitos e violação da dignidade e da autonomia feminina (ludibriamento, chacota e humilhações)^(5)^.

**Violência física:** qualquer procedimento que cause dor ou dano físico sem recomendações baseadas em evidências. São exemplos: privação de alimentos, privação de movimentos, cesariana eletiva e sem indicação clínica, manobra de Kristeller^(5)^.

**Violência Sexual:** violação da intimidade ou pudor, incidindo sobre o senso de integridade sexual e reprodutiva, podendo ter acesso ou não aos órgãos sexuais e partes íntimas do seu corpo. Episiotomia, exames de toque invasivos, constantes ou agressivos, lavagem intestinal, cesariana sem consentimento informado são exemplos dessa violência^(5)^.

**Institucional:** ações ou formas de organização que dificultem, retardem ou impeçam o acesso da mulher aos seus direitos constituídos, como omitir ou violar os direitos da mulher durante o período de gestação, parto e puerpério, impedir acesso aos serviços de atendimento à saúde, impedir a amamentação sem justificativa clínica e impedimento de estarem acompanhadas por alguém que seja da sua escolha^(5)^.

**Material:** ações e condutas ativas e passivas com o fim de obter recursos financeiros de mulheres em processos reprodutivos, violando seus direitos já garantidos por lei, em benefício de pessoa física ou jurídica. Caracteriza-se por cobranças indevidas por parte de planos e profissionais de saúde, indução à contratação de plano de saúde na modalidade privativa, sob argumentação de ser a única alternativa que viabilize o acompanhante^(5)^.

**Midiática:** ações praticadas por profissionais através de meios de comunicação, dirigidas a violar psicologicamente mulheres em processos reprodutivos; apologia às práticas cientificamente contraindicadas, com fins sociais, econômicos ou de dominação. Exemplos: apologia à cirurgia cesariana por motivos vulgarizados e sem indicação científica, ridicularização do parto normal, merchandising de fórmulas de substituição em detrimento ao aleitamento materno, incentivo ao desmame precoce^(5)^.

Além da relação de poder entre profissionais e mulheres, que pode interferir no exercício da autonomia da mulher e na preservação da integridade corporal e psicológica, o reconhecimento do direito das mulheres à escolha e recusa informada e de não serem submetidas a intervenções não consentidas é recente, e ainda não faz parte nem da cultura dos profissionais, nem das mulheres. As principais consequências das VO relatadas são: depressão, medo de repetir a experiência, complicações físicas e mortalidade materno-infantil^(5,8)^.

Diante disso, a enfermagem, como uma profissão alicerçada no cuidar e que participa de forma ativa da assistência em todas as fases de atendimento às mulheres no ciclo gravídico-puerperal, é essencial para o reconhecimento e para a ação em prol da diminuição da VO^(8)^.

### Notificar - reconhecer para agir

O Decreto nº 5.099, de 2004, regulamentou, para todo o território nacional, a notificação compulsória dos casos de violência contra a mulher, atribuindo ao MS a coordenação do plano estratégico de ação para a instalação dos serviços de referência sentinela. Ainda naquele ano, a Portaria MS/GM nº 2.406 instituiu o serviço de notificação compulsória de violência contra a mulher e aprovou instrumento, protocolos e fluxo para notificação nos serviços de saúde público e privado^(9)^.

Em 2016, o MS disponibilizou o Instrutivo “VIVA” com orientações para o preenchimento da Ficha de Notificação de Violência Interpessoal e Autoprovocada. Com o objetivo de subsidiar os profissionais que atuam nas unidades/serviços notificadores para um preenchimento mais padronizado dessa ferramenta de coleta de dados, a partir de um conjunto de variáveis e categorias que retratam as violências perpetradas contra grupos populacionais. Destaca-se no instrutivo a exigência legal da notificação de casos de violência contra a mulher, incluindo a VO^(10)^.

Porém, apesar da obrigatoriedade dos profissionais de saúde ou responsáveis pelos serviços públicos e privados pela notificação compulsória, uma das preocupações expressas pelas políticas de saúde públicas é a falta de registro dos casos de violência contra as mulheres em serviços de saúde. Ademais, a Ficha de Notificação Individual (FIN) não tem nenhum espaço para identificação direta do indivíduo que cometeu o ato violento. Diferente do Boletim de Ocorrência, cujo objetivo é a instauração de inquérito policial para apuração criminal, os dados registrados do provável agressor têm a finalidade de gerar dados epidemiológicos que sirvam de base para estratégias de prevenção e rastreamento, com foco nos cenários de maior risco^(10)^.

A notificação do agravo nada tem a ver com denúncia e outros procedimentos policiais ou de ordem jurídica. A notificação é uma dimensão da Linha de Cuidado para Atenção Integral à Saúde de Crianças, Adolescentes e suas famílias em Situação de Violência, que prevê acolher, atender, promover cuidados profiláticos, o tratamento, o seguimento na rede de cuidado e a proteção social, além das ações de vigilância, prevenção das violências e promoção da saúde e da cultura da paz^(10)^. A FIN não apresenta campo específico para identificação de casos de VO, entretanto o campo 56 da FIN (Figura 1) dá opção de registrar os vários tipos de agressões ocorridas. No campo 52 (Figura 2) é possível registrar o local de ocorrência como “09” (outro), e os detalhes relacionados ao ciclo gravídico-puerperal podem ser anotados no espaço reservado para informações complementares (Figura 3)^(10)^.

**Figura 1:** Campo 56

**Figura 2:** Campo 52

**Figura 3:** Informações Complementares

*Fonte: VIVA - Instrutiva notificação de violência interpessoal e autoprovocada (2024).*

Diante do exposto, para que as informações realizadas em notificações auxiliem no planejamento e implementação de políticas públicas de enfrentamento eficazes e para que se atinja um preenchimento completo e correto da ficha de notificação, é imprescindível que as informações sejam válidas, atuais e completas, e que haja conhecimento dos profissionais sobre os tipos de violência e sobre a correta identificação dos casos suspeitos. Esses dados e informações são essenciais para a qualidade do atendimento prestado pelas equipes de saúde, o gerenciamento das unidades e serviços, o planejamento das ações e políticas de saúde, e para a produção do conhecimento e controle social, pois a notificação da violência constitui o primeiro passo no caminho das ações de controle desse agravo^(8)^.

Ademais, sabemos que somente o reconhecimento da VO como desafio a ser enfrentado não encerra a discussão. A notificação da VO é apenas um passo dentre tantas ações necessárias para a diminuição de todas as violências contra a mulher. A solução depende de diversas estratégias, como as ações educativas em saúde^(8)^. Um exemplo de ação é o curso no modelo *Massive Open Online Course* (MOOC) para capacitação de profissionais da saúde acerca da VO, desenvolvido no programa de Mestrado Profissional da Universidade Federal do Paraná (UFPR), a ser disponibilizado para trabalhadores e trabalhadoras do Complexo Hospital de Clínicas da UFPR, administrado pela Empresa Brasileira de Serviços Hospitalares.

## CONSIDERAÇÕES FINAIS

Todas as mulheres têm direito a um parto sem violência, com respeito, com assistência e escolha informada e com condutas baseadas nas melhores evidências científicas. É necessário falar sobre VO, sobre seus riscos e notificar para romper com essa forma de assistência que coloca a mulher em posição de inferioridade, submissão e não oferece o cuidado e a atenção de forma segura e humanizada.

Reflexões não são conclusivas; ao contrário, são pontos de partida para analisarmos temas que podem ser contemplados e debatidos a partir de vários pontos. Nesta reflexão, consideramos que, para se alcançar a igualdade de gênero, ponto principal do ODS 5, a notificação da VO deve ser amplamente estudada e discutida. Assim como a notificação tem sido uma importante ferramenta para o reconhecimento de outras violências praticadas contra a mulher, também deve ser uma ferramenta utilizada para o reconhecimento da VO.

A subnotificação dos casos de VO compromete direta e negativamente o alcance dos ODS - especificamente no que se refere ao objetivo 5, “Igualdade de Gênero”, mas também aos objetivos 3, “Saúde e Bem-estar”, e 10, “Redução das Desigualdades”.

Profissionais de enfermagem que realizam a assistência obstétrica devem realizá-la de maneira segura e humanizada, livre de práticas invasivas e desnecessárias. Devem prover meios de educação às gestantes e familiares sobre esta temática, direitos e instrumentos para registros em caso de violação destes. Espera-se que este artigo contribua para que profissionais da enfermagem que atendem às mulheres no ciclo gravídico-puerperal procurem compreender a notificação de casos de VO. Notificar é uma forma de dar voz a mulheres que sofrem caladas uma violência institucional naturalizada e que, por vezes, passa despercebida.
