# Supplementary material for: ERRATUM
Source: Rev Bras Enferm. 2025 Dec 8;78(6):e2025n6e09. doi: 10.1590/0034-7167.20257806e09 (PMC12707525; doi:10.1590/0034-7167.20257806e09)
Supplement: Translation

# Translation: ERRATA

**DOI:** 10.1590/0034-7167.20257806e09pt

No artigo “Obstetric violence: reflection on reporting to achieve sustainable development goals”, com número DOI: https://doi.org/10.1590/0034-7167-2024-0523pt, publicado no periódico Revista Brasileira de Enfermagem, 2025;78(3):e20240523, na autoria:

Onde se lê:

**Isabele Melo Martins^II^**

**ORCID: 0000-0002-4455-0499**

Lê-se:

**Isabelle Melo Martins^II^**

**ORCID: 0000-0002-4455-0499**
